# Very Late Presentation of Csf Rhinorrhea after 45 Years of Head Trauma: A Case Report

**DOI:** 10.22038/ijorl.2021.41590.2357

**Published:** 2021-03

**Authors:** Kulwinder-Singh Sandhu, Laika Chopra, Jagdeepak Singh, Sania Arya

**Affiliations:** 1 *Department of Otorhinolaryngology, Ram Lal Eye and ENT Hospital, Government Medical College, Amritsar, Punjab.*

**Keywords:** Cerebrospinal fluid, Head injury, Nasal endoscopy, Rhinorrhea

## Abstract

**Introduction:**

Very late presentation of cerebrospinal fluid rhinorrhea is quite rare with some factors like brain shrinkage with age and ethmoidal growth fracture that lead to leakage from fracture site.

**Case Report:**

We present a 70 years old man diagnosing with CSF rhinorrhea, 45 years after head injury with metallic rod. Treatment of cerebrospinal fluid leakage was performed successfully by endoscopic intranasal repair.

**Conclusion:**

The cerebrospinal leak can occur years after the traumatic head injury even though patient may not had cerebrospinal fluid leakage at the time of trauma. The physician should be aware of the possibility of very late presentation of the cerebrospinal leakage even after years of traumatic head injury.

## Introduction

In 200 BC, Galen was the first to describe the cerebrospinal fluid rhinorrhea (1). It is defined as the leakage of cerebrospinal fluid through the nose and is associated with the presence of a connection between the cranial and nasal cavity and a break in the durameter (2). Head injury with cranial fractures leads to 80% of all the cases of cerebrospinal leakage (3). 

## Case Report

A 70 years aged male came to ENT outpatient department of Government Medical College, Amritsar in April 2018 with chief complaint of clear watery discharge from the right nasal cavity for 3 months which got aggravated while bending forward and did not get sniffed back. History of nasal blockage, headache, fever, post nasal discharge and fits was not present.

Patient gave alleged history of head injury in year 1973 over the vertex with metallic rod following which he fell down facing forward. According to patient, he was unconscious for 6 days and remained hospitalized for the same. When he regained the consciousness, he complained of decreased vision in the left eye with no complaint regarding ear, nose or throat. As per the patient, he underwent surgery (craniotomy) after 1 month of trauma in a private hospital at Amritsar and was discharged in satisfactory condition. No record regarding the hospital stay is available with the patient.

**Fig 1:**
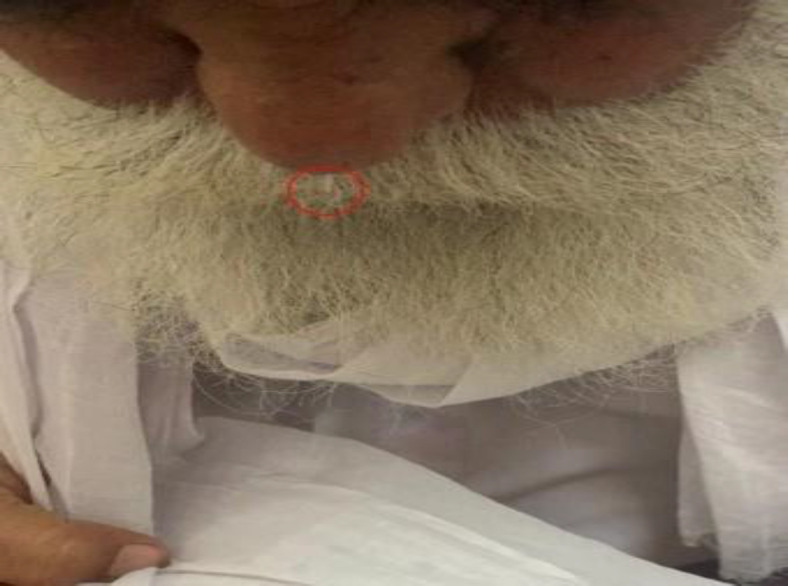
Circle depicts cerebrospinal fluid leakage from right nasal cavity

Patient remained asymptomatic for 45 years. In January 2018, he noticed the leakage of clear watery discharge from the right nasal cavity. According to the patient, he is hypertensive but not on regular treatment for the same. Local examination revealed clear watery discharge coming from the right nasal cavity that increases on bending forward (Fig.1). 

The collected fluid was biochemically analysed that showed cerebrospinal fluid. Beta transferrin test was positive. Eye examination revealed 6/36 vision in right eye and 6/24p vision in left eye with presence of immature senile cataract in both the eyes. Eye movements were within the normal limit. On fundoscopy, there was grade 2 hypertensive retinopathy with attenuation of retinal vessels in both the eyes. 

**Fig 2:**
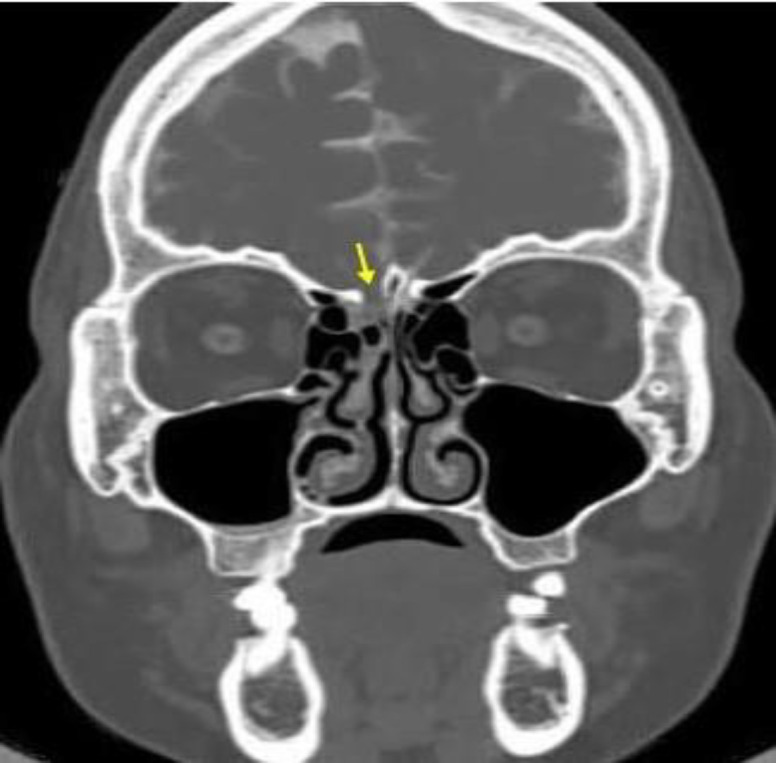
Arrow depicts bony defect in the cribriform plate of ethmoid bone on right side

Both the nasal cavities were normal on anterior rhinoscopy. Diagnostic nasal endoscopy showed clear watery fluid coming out from the roof i.e medial to the attachment of middle turbinate of the right nasal cavity that increased on valsalva manoeuver. NCCT scan nose and PNS confirmed the diagnosis with reporting of ethmoidal bony defect in the cribriform plate on right side, and parietal bone on left side (Fig.2).

Under general anaesthesia, repair of cerebrospinal fluid leakage was performed endoscopically through endonasal route. Steps followed were:

Oropharynx was packed with ribbon roll. Nasal cavities were packed using decongestant packs. After 10 minutes, the middle turbinate, uncinate process and mucosa was infiltrated with 2% xylocaine with adrenaline. Uncinectomy and ethmoidectomy was done using 0 degree 4 mm endoscope. Middle turbinate was excised in its anterior two-third. The skull base was exposed and the site of CSF leak was identified. There was a ethmoidal bony defect of about 8 mm in diameter in the area of cribriform plate. The duramater was freshened along the edges of the defect. Fascia lata graft of size approximately 4 cm x 4 cm was harvested from the right thigh of the patient under all aspetic precautions. Steps for reconstruction of fascia lata were: 5cm long vertical incision was given over the lower lateral side of the right thigh 5cm above the flexed knee joint. Subcutaneous tissue, fat was retracted and white glistening fascia was identified and harvested for the procedure. Incision site was closed with 2-0 silk non absorbable sutures and sterile dressing done.

A U shaped mucoperichondrial flap was raised from the right sided nasal septum with its base towards the dural defect. After harvesting a piece of cartilage (approximately 9 mm in size) from the nasal septum, it was placed over the dural defect. Fascia was inserted (as double layer) to be finally tucked in between the dura and bone. Mucoperichondral flap was rotated and placed over the fascia covering the dural defect as reinforcement over which gel foam. The nasal cavity was packed using merocel. After the hemostasis was ensured and throat suction done, throat pack was removed. Recovery from anaesthesia was uneventful.

Merocel was removed after 72 hours. Postoperatively patient’s head was kept elevated, regular blood pressure monitoring was done and antitussives and laxatives were given. He was discharged on 10th postoperative day. Similar instructions were given at the time of discharge. No recurrence has been evident at his follow up visits till date.

## Discussion

Cerebrospinal fluid rhinorrhea after a traumatic skull fracture was first described by Bidloo the Elder in 1745 (4). Cerebrospinal fluid is a modified tissue fluid around brain and spinal cord acting as a buffer against sudden jerk (5). It is produced by the specialized ependymal cells in the choroid plexuses of the lateral, third and fourth ventricles of brain. The etiology of cerebrospinal leak includes the traumatic as well as non-traumatic causes. 80-90% of all the causes of CSF leak are due to head injury (6).

The cerebrospinal leakage from the anterior cranial fossa is the most common site as the bone of the anterior skull is thin and densely adherent to the dura. So, the possibilities of dural tears are more common (7).

In general, more than half of the cerebrospinal leaks present immediately within 48 hours of the trauma or shortly after the injury i.e. within 3 months (8). Very late onset of the cerebrospinal leak is quite rare with limited studies available.

More than 60 years ago, a case series of 84 patients of cerebrospinal rhinorrhea was published and concluded that only 3 patients presented after more than 3 months of their initial injury (9). Another case series was published recently that included 51 traumatic cerebrospinal fluid leaks and showed that only 8 patients presented with cerebrospinal leakage within months to years after the initial injury (10).

The reason for the very late onset of cerebrospinal fluid leak may include:

- Brain shrinkage caused by age leading to cerebrospinal fluid leaking site re-opening.

- Ethmoidal growth fracture that lead to cerebrospinal leakage from the fracture site (11). Along with it, predisposing factors like obesity and hypertension increases the risk of delayed cerebrospinal rhinorrhea.

The diagnosis mainly requires a clinical examination; biochemical analysis of collected fluid along with radiological evaluation i.e high resolution computed tomography (HRCT) of nose and PNS, CT cisternography and magnetic resonance imaging (MRI) with intrathecal contrast or cisternography for the description of the exact size and site of the defect (12).

Treatment of cerebrospinal rhinorrhea comprises of conservative or surgical management. Surgical management can be done by three approaches ie intracranial, extracranial and transnasally. Nowadays, the transnasal endoscopic method is the preferred method for the repair ofcerebrospinal fluid leakage with overall 90% success rate (11). 

## Conclusion

The cerebrospinal leak can occur years after the traumatic head injury even though patient may not had cerebrospinal fluid leakage at the time of trauma. The physician should be aware of the possibility of very late presentation of the cerebrospinal leakage even after years of traumatic head injury.
